# Suramin Inhibits Chikungunya Virus Replication by Interacting with Virions and Blocking the Early Steps of Infection

**DOI:** 10.3390/v12030314

**Published:** 2020-03-17

**Authors:** Irina C. Albulescu, Leonie White-Scholten, Ali Tas, Tabitha E. Hoornweg, Salvatore Ferla, Kristina Kovacikova, Jolanda M. Smit, Andrea Brancale, Eric J. Snijder, Martijn J. van Hemert

**Affiliations:** 1 Department of Medical Microbiology, Leiden University Medical Center, 2333 ZA Leiden, The Netherlands; i.c.albulescu@uu.nl (I.C.A.); Leonie.White-Scholten@hollandbio.nl (L.W.-S.); a.tas@lumc.nl (A.T.); K.Kovacikova@lumc.nl (K.K.); E.J.Snijder@lumc.nl (E.J.S.); 2 Department of Medical Microbiology, University Medical Center Groningen, University of Groningen, 9713 GZ Groningen, The Netherlands; t.e.hoornweg@uu.nl (T.E.H.); jolanda.smit@umcg.nl (J.M.S.); 3 School of Pharmacy and Pharmaceutical Sciences, Cardiff University, Cardiff CF10 3NB, UK; FerlaS1@cardiff.ac.uk (S.F.); BrancaleA@cardiff.ac.uk (A.B.)

**Keywords:** CHIKV, alphavirus, antiviral, suramin, E2 envelope protein, attachment, fusion, drug repurposing

## Abstract

Chikungunya virus (CHIKV) is a mosquito-transmitted alphavirus that can cause a debilitating disease that is primarily characterized by persistent joint pain. CHIKV has been emerging globally, while neither a vaccine nor antiviral medication is available. The anti-parasitic drug suramin was previously shown to inhibit CHIKV replication. In this study we aimed to obtain more detailed insight into its mechanism of action. We found that suramin interacts with virions and can inhibit virus binding to cells. It also appeared to inhibit post-attachment steps of the infection process, likely by preventing conformational changes of the envelope glycoproteins required for fusion and the progression of infection. Suramin-resistant CHIKV strains were selected and genotyping and reverse genetics experiments indicated that mutations in E2 were responsible for resistance. The substitutions N5R and H18Q were reverse engineered in the E2 glycoprotein in order to understand their role in resistance. The binding of suramin-resistant viruses with these two E2 mutations was inhibited by suramin like that of wild-type virus, but they appeared to be able to overcome the post-attachment inhibitory effect of suramin. Conversely, a virus with a G82R mutation in E2 (implicated in attenuation of vaccine strain 181/25), which renders it dependent on the interaction with heparan sulfate for entry, was more sensitive to suramin than wild-type virus. Using molecular modelling studies, we predicted the potential suramin binding sites on the mature spikes of the chikungunya virion. We conclude that suramin interferes with CHIKV entry by interacting with the E2 envelope protein, which inhibits attachment and also interferes with conformational changes required for fusion.

## 1. Introduction

Chikungunya virus (CHIKV) is a re-emerging alphavirus that is transmitted by *Aedes sp.* mosquitoes and has caused several large outbreaks in the past 15 years. Before 2013 CHIKV was circulating mainly in Africa and Asia [1], but following its introduction into the Caribbean it has now become endemic in Latin America as well [2]. Acute CHIKV infection is associated with fever, rash, muscle pain, and general malaise. Furthermore, the virus often causes a debilitating joint pain that can last for months to years [3]. Prophylactic or therapeutic treatment for CHIKV infections is still not available on the market, and vector control measures do not provide the ultimate solution [4]. Although progress is being made in CHIKV vaccine and antiviral drug development [3,5,6,7], we would still be poorly prepared in the face of a new CHIKV epidemic.

We and others have previously shown that the antiparasitic drug suramin inhibits CHIKV replication by targeting an early step in the viral replication cycle [8,9,10]. Moreover, the compound also inhibits CHIKV replication in a mouse model [11]. These findings show the potential for the (off-label) therapeutic use of suramin for the treatment of chronic chikungunya fever, and possibly also for its prophylactic use during severe CHIKV outbreaks.

CHIKV has a 11.8-kb single-stranded (ss) RNA genome of positive polarity, which is capped, poly-adenylated and packaged into an icosahedral nucleocapsid that is surrounded by an envelope containing 80 projections (spikes), each consisting of three E1-E2 (envelope proteins) heterodimers [12]. The E2 protein is involved in the interaction with the host cell and therefore is an important determinant of pathogenicity, cellular tropism [13,14], and immunogenicity [15,16]. The viral replication cycle begins with attachment of the virion to the cell surface through interactions with glycosaminoglycans, such as heparan sulfate [17]. Subsequent binding to a receptor, like the recently identified Mxra8 protein [18] will lead to uptake of the virion via receptor-mediated endocytosis [19]. Endosomal acidification causes structural rearrangements in the virion that induce E1 protein-mediated fusion of the viral envelope with the endosomal membrane [20]. This leads to nucleocapsid release and its disassembly in the cytoplasm to liberate the RNA genome. Subsequently, the genome is translated into a polyprotein that is processed into the non-structural proteins nsP1 to 4 that (together with host factors) assemble into membrane-associated replication and transcription complexes (RTCs). The structural proteins (capsid, E3, E2, 6k/TF and E1) are expressed from a subgenomic RNA in the form of a second polyprotein. After autoproteolytic release of the capsid protein, the remainder of the structural polyprotein traffics through the secretory pathway, during which it is cleaved by host cell proteases and undergoes post-translational modifications like glycosylation. Ultimately, the mature envelope proteins will reach the plasma membrane. Here, interaction between nucleocapsids and the cytoplasmic sides of the envelope proteins are essential for the budding process and the formation of new virus particles [21].

The work presented here provides insight into the mode-of-action of suramin, through specific analysis of virus binding and fusion, and by selecting and characterizing suramin-resistant CHIKV variants, which contained N5R and H18Q mutations in the envelope protein E2. Moreover, we found that a virus with the G82R mutation in E2, which renders the virus dependent on heparan sulfate binding for infectivity and was implicated in attenuation of vaccine strain 181/25 [13], was more sensitive to suramin than wild-type CHIKV. Molecular docking studies provided more insight into suramin’s inhibitory activity, since the compound was predicted to bind to virus particles at positions that could interfere with conformational changes in the envelope proteins that need to occur during entry.

## 2. Materials and Methods

### 2.1. Cells, Compounds, and Viruses

Vero E6 and BHK21 cells were grown in DMEM or BHK medium (G-MEM BHK-21 with 10% Tryptose phosphate broth and 10 mM HEPES), respectively, supplemented with 8% fetal calf serum (FCS) and penicillin/streptomycin. BS-C-1 cells were cultured as previously described [22]. Suramin, chloroquine and ammonium chloride were purchased from Sigma and ^3^H-suramin from Hartmann Analytic. CHIKV LS3 (KC149887) and Semliki Forest virus strain SFV4 (KP699763.1) were launched from full-length cDNA clones. All studies with live CHIKV were performed in biosafety cabinets in BSL-3 facilities.

### 2.2. Preparation of ^35^S-labeled Viruses and Purification of Virus Stocks

^35^S-labeled CHIKV and SFV were produced in Vero E6 cells as described before [23]. To remove non-incorporated labels, the cell culture supernatant was subjected to pelleting through a sucrose cushion by ultracentrifugation in a SW41 Ti rotor (at ~200,000 g for 2 h). Virus pellets were resuspended in 1xTESV buffer (20 mM Tris-HCl pH 7.4, 1 mM EDTA, 100 mM NaCl), before aliquoting and storage at −80 °C.

### 2.3. Virus Attachment Assay

To study virus binding, ^35^S-CHIKV or ^35^S-SFV samples (1 × 10^4^ counts per minute, CPM) were incubated with Vero E6 cells for 1 h at 4 °C, in the presence or absence of suramin, followed by washing and lysis in 4x dye-free Laemmli sample buffer (LSB). The amount of bound radioactivity in samples was quantified by liquid scintillation counting with a Beckman-Coulter LS6500 Multi-Purpose scintillation counter and Ultima Gold™ scintillation liquid. The binding of fluorescently labeled CHIKV (DiD-CHIKV) to BS-C-1 cells was assessed by fluorescence microscopy, as previously described [22].

### 2.4. Bulk Fusion Assay

Pyrene-labelled CHIKV and liposomes containing phosphatidylcholine (PC), phosphatidylethanolamine (PE), sphingomyelin and cholesterol in a molar ratio of 1:1:1:1.5 were used in a bulk fusion assay as previously described [19].

### 2.5. ^3^H-Suramin-Virus Binding Assay

Purified virus particles prepared in 1xTESV buffer (containing only trace amounts of protein from the culture medium) were incubated with 0.5 × 10^6^ CPM of ^3^H-suramin, for 1 h at 37 °C. The unbound suramin was removed by gel-filtration using P30-Microbiospin columns (BioRad, Berkeley, CA, USA) according to the manufacturer’s instructions; virions in the flow-through were lysed in dye-free LSB and bound radioactive suramin was quantified by scintillation counting as described under Section 2.3.

### 2.6. Reverse Genetics

The mutations listed in Table 1 were introduced into the pMALS2L (G588A, A979G, G980A, T5645C) and pMALS2R (A8554G, C8595A) plasmids [24] using QuickChange site-directed mutagenesis (Stratagene, La Jolla, CA, USA). Subsequently, single or combined mutations were transferred to the CHIKV LS3 plasmid by using unique BamHI, XmaI, AgeI, XhoI and SfiI restriction sites. The reverse-engineered mutant CHIKV mutants were launched via in vitro transcription (AmpliScribe™ T7 High Yield Transcription Kit, Lucigen, Middleton, WI, USA) and RNA transfection of BHK-21 cells as described previously [24]. After 24 to 48 h, when extensive cytopathic effects (CPE) had occurred, the virus-containing supernatants were harvested and used to produce passage 1 virus stocks on Vero E6 cells, which were used in subsequent experiments. These virus stocks were verified by Sanger sequencing of the full genome to confirm the presence of the introduced mutations and absence of other mutations. Only in the case of G980A (in nsP1) we observed rapid reversion to the wild-type genotype.

### 2.7. RNA Isolation and RT-qPCR

Total cellular RNA was isolated by lysing the cells in LiDS/LET as previously described [24] or in TriPure reagent according to the manufacturer’s (Invitrogen, Waltham, MA, USA) instructions. To measure genome copy numbers an internally controlled TaqMan quantitative RT-PCR assay was used [24].

### 2.8. Cytopathic Effect (CPE) Reduction Assay

CPE reduction assays were basically performed as previously described [8] with some modifications. VeroE6 cells were seeded in 96-well clusters at a density of 5000 cells/well the day before infection. The following day, the medium was replaced with serial dilutions of suramin in EMEM/2% FCS and cells were infected with CHIKV at an MOI of 0.05. The incubation period was 72 h for CHIKV mutants S4.1, S4.3, S5 and 96h for wt CHIKV and mutants S2.1, S2.3, S3, S7, S8 and S9, or 120 h for mutants S2.2 and G82R. Suramin-treated uninfected cells were included as controls to exclude potential cytotoxic/cytostatic effects of the compound treatment. After performing a colorimetric viability assay with MTS reagent (Promega, Leiden, The Netherlands) for 2 h at 37 °C, 30 µl/well of 37% formaldehyde was added and absorption was measured at 450 nm using an EnVision Multilabel Plate Reader (PerkinElmer, Waltham, MA, USA). The EC_50_, defined as the concentration of suramin required to inhibit virus-induced CPE by 50% was determined by non-linear regression analysis using GraphPad Prism v8.0.

### 2.9. Plaque Number Reduction Assay

To study virus entry, Vero E6 cells were incubated with approximately 100 plaque forming units (PFU) of CHIKV for 1 h at 37 °C in the presence of a range of suramin concentrations. After removing the inoculum, the cell monolayer was washed and overlay medium containing 1.2% Avicel RC-581 (FMC BioPolymer, Philadelphia, PA, USA) in DMEM, 2% FCS, 25 mM HEPES, and penicillin/streptomycin was added. After three days, the cell monolayers were fixed with 3% formaldehyde in PBS solution and plaques were stained and counted. To study attachment, suramin treatment and virus uptake were done for 1 h at 4 °C, and 1000 PFU were used in order to detect approx. 100 plaques in the untreated wells, as low temperature diminished virus binding.

### 2.10. Molecular Modelling

Molecular Operating Environment (MOE) 2018.10 [25] and Maestro [26] software was used. The CHIKV E2-E1 glycoprotein heterodimer (PDB ID 3N42) and trimeric complex (PDB ID 3J2W) were preprocessed using the Schrödinger Protein Preparation Wizard by assigning bond orders, adding hydrogens, and performing a restrained energy minimization of the added hydrogens using the OPLS_2005 force field. The missing residues of E2 (1–6) were manually introduced and the downstream docking processes are described in more detail in Appendix A. The electrostatic potential surface was obtained using the Surfaces and Maps tool in MOE after splitting the molecule in multiple chains. Figures were prepared with MOE.

### 2.11. Statistics

**Figure 1:**
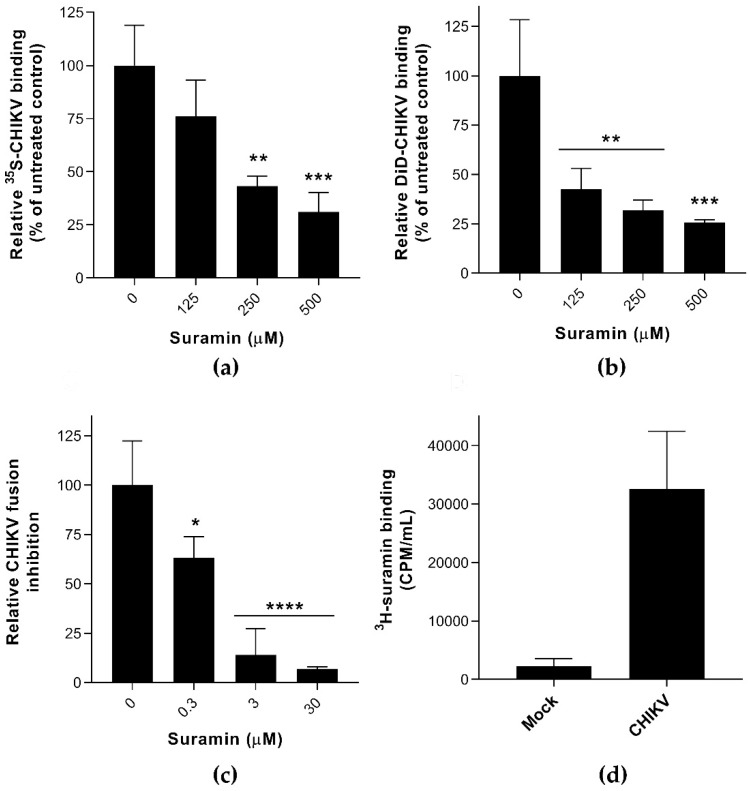
The effect of suramin on the early events of Chikungunya virus (CHIKV) infection (**a**) The binding of ^35^S-labelled CHIKV (1 × 10^4^ CPM) to Vero E6 cells in the presence or absence of suramin was determined at 4 °C by scintillation counting of remaining radioactivity in cellular lysates obtained after extensive washing (average +/− SD; *n* = 3). (**b**) Binding of fluorescently (DiD)-labeled CHIKV to suramin-treated BS-C-1 cells, analyzed by fluorescent microscopy, in the presence of increasing compound concentrations. (**c**) Fusion of pyrene-labeled CHIKV in a bulk fusion assay with liposomes, triggered by lowering the pH, in the presence of increasing suramin concentrations (*n* = 5 and 3, for untreated and treated samples, respectively). (**d**) Binding of ^3^H-labeled suramin (5 × 10^5^ CPM) after a 1-h incubation at 37 °C to CHIKV (purified virus was used to exclude interference by serum proteins). The control used in this assay was culture medium from uninfected cells that was treated the same way as when purifying virus (*n* = 3). The data represent the means ± the SD and significant differences are indicated with * (**** *p* < 0.001, *** *p* < 0.005, ** *p* < 0.01, * *p* < 0.05 and ns as not significant).

GraphPad Prism 8 was used as previously described [8,23] for EC_50_ determination by non-linear regression. The statistical analysis was performed with one-way ANOVA using Dunnett’s multiple comparison test (Figure 1a–c).

## 3. Results and Discussion

### 3.1. Suramin Inhibits Viral Attachment and Fusion by Interacting with the Chikungunya Virion

Several experiments were carried out to analyze the impact of suramin on the early events of infection, i.e., virus attachment, internalization, and fusion. ^35^S- or DiD-labeled CHIKV [22] were used in virus binding assays, in the presence or absence of suramin, at 4 °C. Active endocytosis does not occur at temperatures below 18 °C [27] and therefore suramin was expected to remain in the extracellular environment. Virus attachment to the cell surface probably involves electrostatic interactions with the GAGs or other plasma membrane factors (receptors, adhesion molecules) and infections can be synchronized by placing cells at 4 °C. By directly measuring the amount of radioactively- or fluorescently-labeled virus in the presence of increasing concentrations of compound, we found that suramin inhibited CHIKV attachment (Figure 1a,b) in two distinct cell lines and with two different experimental readouts. Binding of ^35^S-labelled SFV was even more strongly inhibited by suramin (supplemental Figure S1a), which is in line with suramin’s previously observed lower EC_50_ for SFV compared to CHIKV [8]. In addition, suramin (30 μM) completely inhibited pyrene-labeled CHIKV from fusing with liposomal membranes at pH ~5.5 in the absence of host proteins, as shown in Figure 1c [19]. Because suramin is known to bind to positive charges on the surface of proteins, we suspect that suramin binds directly to the envelope proteins, thereby preventing the conformational changes that are required for fusion. In contrast to Ho et al., who studied the effect of suramin on surface-expressed envelope proteins in insect cells that were subsequently triggered to fuse at low pH, we studied the effect of suramin on membrane fusion in the context of whole virions. The ~100-fold lower EC_50_ in the fusion assay (Figure 1c) compared to the binding assay (Figure 1a) makes it tempting to conclude that suramin inhibits fusion much more than binding, but these differences might very well be due to the very different experimental setup of the (in vitro liposome-based) fusion and the (cell-based) binding assay.

To confirm the interaction of suramin with virions, we incubated purified CHIKV (lacking serum proteins as these are known to strongly bind suramin; [28]) with ^3^H-labeled suramin. Compared to a control supernatant from mock-infected cells (which bound 3000 CPM of suramin) purified CHIKV bound over 30,000 CPM of ^3^H-suramin (Figure 1d), confirming that the compound did interact with virus particles. ^3^H-suramin also interacted with SFV (Figure S1c), and more specifically with the (native) E proteins on the surface of intact virions, since treatment with proteinase K or heat denaturation severely decreased the quantity of bound radiolabeled suramin. For other viruses like enterovirus A71 (EV-A71) and human immunodeficiency virus 1 (HIV-1) it was also found that suramin blocked their access to cellular receptors by directly interacting with virions [29,30].

Surprisingly, when SFV particles were pre-attached to cells (at 4 °C, in the absence of compound) and treated with suramin at 37 °C, only a minor non-significant inhibition of virus uptake was observed, even at high suramin concentrations (Figure S1b). Hence it is possible that at physiological temperature, pre-attached viruses cannot be displaced from their receptors by the compound, implying that the receptor- and suramin-binding sites overlap or the entry process might be too fast to be inhibited by suramin. When evaluating these direct binding studies, it should be noted that it remains unclear what fraction of the bound virions will actually lead to a productive infection. 

The inhibitory effect of suramin on the binding of virus particles to the cell surface could be due to its direct interaction with virions and/or with cellular receptors. The direct binding assays with radiolabeled or fluorescently labeled viral particles clearly showed that suramin inhibits binding/fusion of both CHIKV and SFV (Figure 1 and Figure S1). This is in contrast with our earlier study [8], in which we concluded that suramin inhibits an early step, but not attachment. In that study, we relied on an RT-qPCR assay to quantify cell-bound CHIKV. However, this is an indirect measurement and the majority of the detected RNA molecules do not represent infectious particles, as we and others found that genome copy to PFU ratios of commonly used virus stocks were generally over 1000:1 [31,32]. At least in part, this is due to the fact that virus stocks are generally harvested when extensive cytopathic effect (CPE) has occurred, which leads to the release of viral RNA not associated with infectious virus particles, e.g., in the form of naked RNA, nucleoprotein complexes or nucleocapsids. Therefore, we no longer consider RT-qPCR an appropriate assay to study CHIKV binding when standard, non-purified CHIKV specimens are used.

To evaluate whether the effect of suramin on CHIKV binding could be reliably measured by an improved RT-qPCR-based assay, we have used a PEG-precipitated virus stock to improve the CHIKV RNA to PFU ratio. After titration on Vero E6 cells, the ratio ranged from 7632:1 in the non-purified stock to 68:1 in the PEG-precipitated stock. Clearly, even after this procedure, the number of CHIKV RNA molecules still strongly outnumbers the number of infectious particles. Subsequently, we repeated the qPCR-based measurement of virus binding at 4 °C in the presence and absence of suramin (Figure S2), for both non-purified and PEG-precipitated virus stocks. While for the former there was an unexpected increase in detected CHIKV RNA in the presence of suramin, the PEG-precipitated stock showed a slight decrease, confirming that the quality of the virus stock strongly influences the experimental outcome and conclusions.

### 3.2. Suramin-Resistant CHIKV Variants Acquired Mutations in the Envelope Protein E2

Repeated passaging of CHIKV in the presence of increasing suramin concentrations that do not fully inhibit replication (from 25 up to 300 µM) yielded variants that could grow to titers above 10^5^ PFU/mL in the presence of 150 µM suramin. By passage 5 (P5) and passage 7, drug concentrations of 150 and 300 µM, respectively, were tolerated, concentrations that reduced wild-type virus titers by at least 2 logs [8]. Genotyping of the P5 virus revealed the presence of three nonsynonymous mutations in the CHIKV nsP1, nsP3 and E2 proteins. Two passages later (P7), the suramin-resistant variant had acquired several additional mutations in the same 3 proteins (Table 1).

To pinpoint which of these mutations is/are responsible for the suramin-resistant phenotype, they were all reverse engineered into our CHIKV full-length cDNA clone [24], either alone or in combination. For each of the reverse-engineered viruses, the plaque phenotype and sensitivity to suramin were determined and for several mutants the growth kinetics were also compared. 

Viruses with the R171Q and opal524R mutations (CHIKV mutants S4.1, S4.3 and S5) were found to produce larger plaques on Vero E6 cells, suggesting they could be linked to cell culture adaptation. Both R171Q and opal524R had been previously reported in CHIKV isolates such as MADOPY1, StBI and StVE (GenBank accession numbers KP003808.1, KP003811.1, and KP003810.1 respectively) which all had been passaged in cell culture prior to sequencing [33]. The mutation opal524R in the nsP3-coding region was reported also by Mounce et al. in the context of resistance selection against the compound DFMO [34], alongside with other nsP mutations. In the case of CHIKV and other alphaviruses (SFV, ONNV), evolutionary pressures have maintained both variants (stop and arginine codon) as it offers a fitness advantage when switching between vertebrate and invertebrate hosts [35,36,37]. The combination of the R171Q and opal524R mutations was also found independently during the selection of CHIKV variants that were resistant to an unrelated compound [38], and they merely appear to reflect adaptation to repeated passaging in mammalian cells (under stress). 

**Figure 2:**
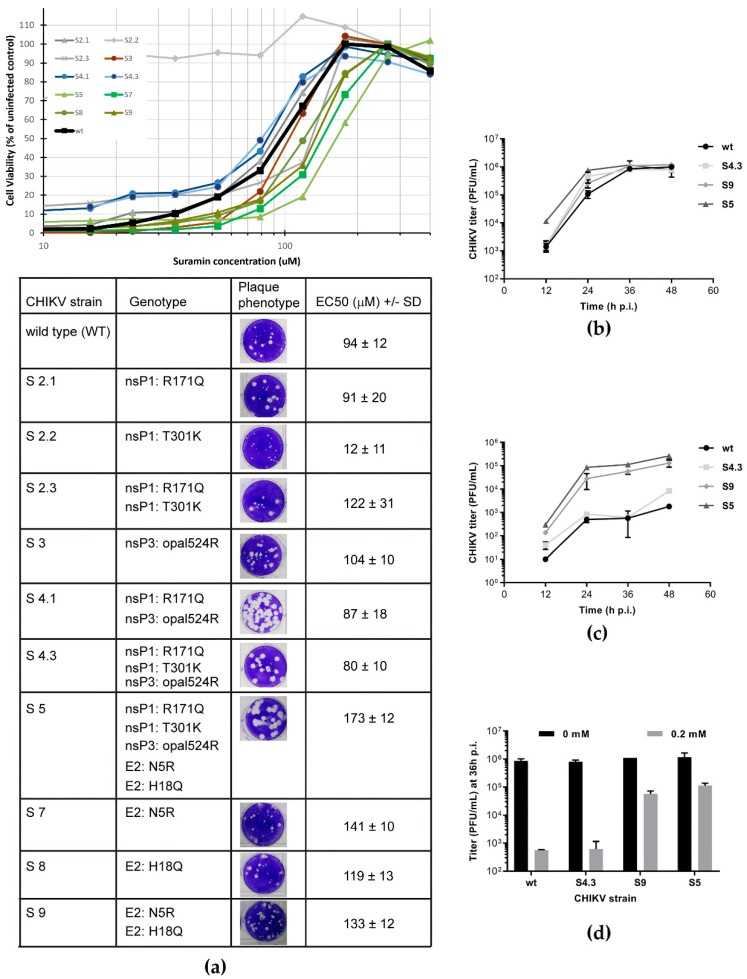
Characterization and suramin sensitivity of reverse engineered CHIKV variants (**a**) Mutations identified in suramin-resistant CHIKV mutants (Table 1) were reverse-engineered (individually or in combinations) into infectious cDNA clone CHIKV LS3. Plaque morphology (in the absence of suramin) and EC_50_ (mean, *n* = 8) for suramin as determined by CPE reduction assay (curves in graph above table) are shown for each of the recombinant viruses. The values were determined from two independent experiments performed in quadruplicate. (**b**,**c**) Replication kinetics of CHIKV mutants S4, S5, S9 and wt virus were compared during infection of Vero E6 cells in the absence (**b**) or presence of 0.2 mM suramin (**c**). At several time-points p.i., culture supernatants were harvested and infectious virus titers were determined by plaque assay (*n* = 2). (**d**) Side-by-side comparison of the 36 h p.i. titers of various mutants and wt virus grown in the absence (N.T., not treated) or presence of 0.2 mM suramin (*n* = 2). All experiments were performed in Vero E6 cells and the data represent mean ± the SD.

In contrast, the T301K mutation in nsP1, causing a small-plaque phenotype, and the two mutations in E2 (N5R and H18Q) that did not alter the plaque phenotype (Figure 2a), have to our knowledge not been previously identified in other isolates. However, the T301 residue was found to be changed to an I in the CHIKV 181/25 vaccine strain, that was attenuated by repeated passaging in MRC-5 cells, and was accompanied by mutations T12I and G82R in E2, C42F in 6K and A404V in E1 [39]. It was later proven that T301I was not related to the attenuated phenotype of the vaccine strain, but that the two E2 mutations were responsible [40].

**Figure 3:**
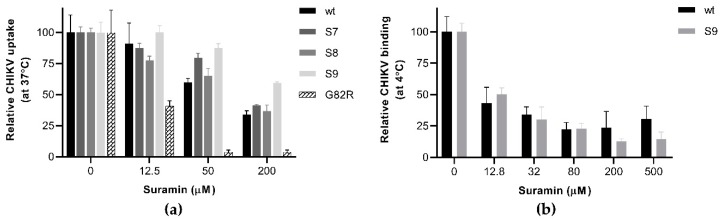
The effect of E2 mutations on suramin-resistance and the early steps of infection (**a**) Virus uptake and infectivity were determined based on a PRNT-like assay. Approx. 100 PFU of wt CHIKV and mutants S7, S8, S9 and G82R were incubated with Vero E6 cells for 1h in the presence or absence of increasing suramin concentrations. Afterwards the inoculum was removed, the monolayers were washed with PBS and overlay medium was added. After a three-day incubation, the cells were fixed and plaques were stained and counted. (**b**) The plaque number reduction assay was used to analyze synchronized attachment of wt CHIKV and variant S9 at 4 °C, in the presence and absence of suramin. After binding for 1 h in the cold, the inoculum and suramin were removed and replaced with overlay medium without suramin, and the rest of the procedure was performed as described under (**a**). The data represent the means ± the SD (*n* = 3).

Reverse engineering of the N5R and H18Q mutations in E2 produced CHIKV variants with an increased tolerance to suramin (Figure 2a), as an increase in EC_50_ was observed when both mutations were present (S5 and S9 in Figure 2a). This suggested that the two E2 mutations cause the suramin-resistant phenotype, identifying E2 as the target of the compound. The N5R mutation alone (S7) already provided suramin resistance, while H18Q (which appeared later in resistance selection) on its own (S8) had a less pronounced effect on resistance. However, as discussed below (Figure 3a), in another more direct assay there is a cumulative positive effect when the H18Q mutation is introduced in a virus that already contains the N5R mutation (S9).

The replication kinetics of relevant mutants, S4.3 (containing mutations in nsPs only), S9 (containing only the E2 mutations), and S5 (containing all mutations) were slightly faster than that of the wt control, reaching slightly higher titers by 24 h p.i., but similar titers by 36 h p.i. (Figure 2b). Despite its faster replication, S4 is as sensitive to suramin treatment as the wt virus, with a 3-log reduction in titers upon treatment with 200 μM suramin. The S9 and S5 mutants, on the other hand, exhibited a titer reduction of only 1 log (Figure 2c,d). These findings indicate that the nsP mutations (the S4 variant) were not involved in suramin resistance and that the E2 mutations alone were responsible for the resistant phenotype. 

### 3.3. The N5R and H18Q Mutations in E2 Enhance CHIKV Entry

To understand how the E2 mutations affect CHIKV infectivity in the presence of suramin, we analyzed the early steps (attachment, uptake, fusion) of infection, using a plaque number reduction assay in which virus and compound were present only during the first hour. We also reverse engineered a virus with a G82R mutation in E2, as this mutation was previously shown to render CHIKV fully dependent on HS binding for infection and is responsible for the attenuated phenotype of CHIKV vaccine strain 181/25 [13,41]. Among all E2 mutant viruses tested, the G82R mutant was extremely sensitive to suramin, as its uptake and infectivity were almost abolished in the presence of 50 µM suramin, whereas this concentration merely caused a 40% reduction for wt virus (Figure 3a). The S7 and S9 mutants were indeed more resistant to suramin than wt virus, as their entry was not significantly inhibited by the presence of 50 µM suramin, while that of wt and S8 was. In the presence of 200 µM suramin, the uptake of wt virus, mutant S7 and mutant S8 was reduced to 40% of that of untreated cells, while the uptake of mutant S9 remained at ~60%, confirming that the presence of both E2 mutations leads to an increased resistance. The binding of mutant S9 at 4 °C, however, was inhibited to the same extent by suramin as that of wt virus, suggesting the N5R and H18Q mutations do not offer resistance to the compound during the attachment step (Figure 3b). This finding was corroborated by a time-of-addition assay where the pre-treatment of cells with suramin inhibited wt and mutant S9 to the same extent. However, if treatment was started at the moment of infection or later, the S9 mutant replicated better than the wt virus (supplemental Figure S3). Therefore, it seems that the two mutations allow the virus to overcome the inhibitory effect of suramin at a post-attachment step, such as fusion.

### 3.4. Molecular Modelling Predicts Suramin to Bind between Two Adjacent E2 Proteins in a Mature Spike

The observations on the involvement of E2 mutations in suramin resistance prompted us to explore the interaction between the viral envelope protein and compound in more detail, by using a molecular docking approach. We employed a molecular docking approach using suramin and the E2 protein structure on its own, in the form of the E1-E2 heterodimer or as present within a mature CHIKV spike. Our initial attempts of docking suramin to an isolated E2 protein structure or to the E1-E2 heterodimer (PDB ID 3N42) revealed that the compound could interact with a region lacking a clear secondary structure (a groove between domains A and C and linked to the flexible N-terminal part of E2). The N5R mutation that was implicated in suramin resistance maps to this flexible region in the N-terminal domain of E2. Based on this predicted binding, the introduction of negative charges at position 6 and 160 (F6D, T160D) were expected to repel the binding of suramin to that area. To test whether this was indeed the case, these mutations were reverse engineered. The recombinant viruses had a plaque size similar to the wt CHIKV, but in CPE reduction assays they were more or equally sensitive to suramin, with EC50 of 54.5 µM (for F6D) and 16.7 µM (for T160D), compared to 55.5 µM of wt CHIKV. Previously Ho et al. have also docked suramin to the E1-E2 heterodimer and predicted that suramin would bind in a region between the two proteins [9]. 

In a more recent publication, a structure for the mature CHIKV spike was obtained by modeling the crystal structures of the E1 and E2 proteins into the cryo-EM image of a CHIKV VLP [42]. Due to this novel information, we now consider the earlier docking studies to be less representative for the natural situation, since in the context of a virion the suggested suramin binding sites would not be exposed in mature spikes, which are formed by E1-E2 heterotrimers (See supplemental Figure S4). Therefore, to refine our understanding of the possible electrostatic interactions between suramin and the surface of CHIKV, we employed a molecular docking approach based on the more relevant model of the envelope heterotrimer. 

**Figure 4:**
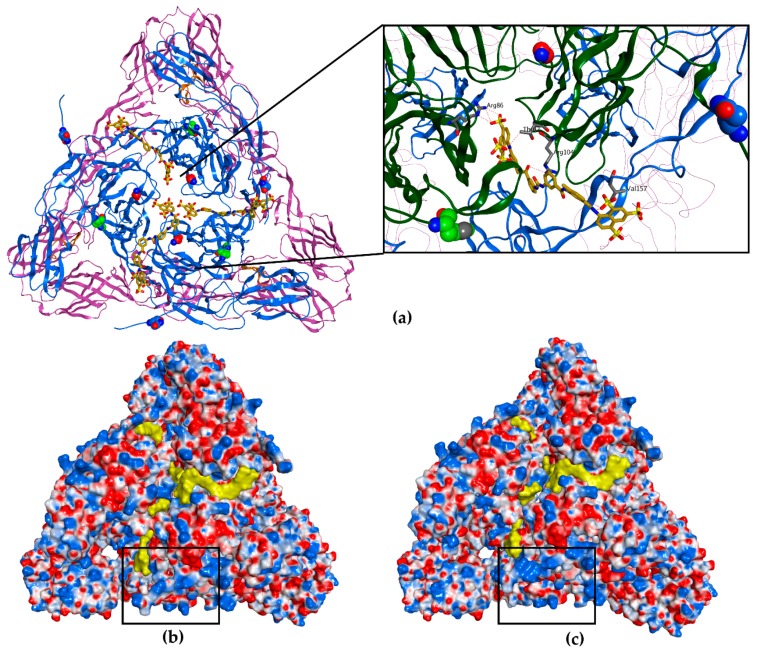
Molecular docking of suramin to a mature CHIKV spike (**a**) The top view of a full wt E1-E2 heterotrimer (PDB ID 3J2W). The E2 proteins are represented as blue ribbon, the E1 as purple ribbon and the fusion loop as orange ribbon; the N5 and H18 residues are represented with carbon atoms in blue and green, respectively, and residue G82 with carbon atoms in red, belong to E2. Suramin is represented in yellow (3 molecules, carbon atoms and molecular surface). The inset (black rectangle) shows a clearer magnified view of the spike core and the positioning of suramin with respect to the residues N5, H18 and G82. (**b**) Electrostatic potential (Coulombic surface coloring) of the heterotrimer of wt CHIKV. The black rectangle marks the N-terminal domain of one E2 protein, where the positive charges are found. (**c**) Electrostatic potential (Coulombic surface coloring) of the heterotrimer of the N5R/H18Q mutant, CHIKV S9. The black rectangle highlights the N-terminal domain of E2 showing an increase in positive charges (blue molecular surface). For presentation purposes, the transmembrane and C-terminal segments of the E1 and E2, which interact with capsid proteins seen in (**a**), were removed.

In this model, suramin (depicted in yellow) interacts (ΔG −96.97 kcal/mol) with a very flexible loop in the N-terminal region of one E2 molecule, while it extends towards the middle of domain A of an adjacent E2 (Figure 4a). Moreover, several suramin molecules are predicted to bind the same spike. Because domain A is involved in receptor recognition, as well as being the target of neutralizing antibodies, it has a pivotal role in the viral replicative cycle [14,16,18,43].

By analyzing the charge distribution on the surface of the CHIKV spike, it became clear that the N-terminal loop of E2 harbors positive charges (K at positions 3 and 10). By acquiring the mutation N5R, there was a clear increase in the positive charges (differences indicated with black rectangles in Figure 4b,c), probably leading to a stronger interaction with suramin and perhaps pulling it away from another area of the spike. Counterintuitively, because one would expect that a resistance mutation would prevent the interaction with suramin, we speculate that the N5R mutation actually attracts the compound thereby changing the binding mode. Perhaps the N5R mutation directs suramin away from the center of domain A, which is known to be involved in receptor recognition and is also the target of neutralizing antibodies [15,18].

According to our molecular modelling prediction, the H18Q mutation, which is located in a region across from the E1 fusion loop, might enhance the effect of the N5R mutation by stabilizing the flexible N-terminal loop to achieve a better interaction with the compound or could cause some other structural changes in the heterotrimer leading to a decreased affinity for suramin. Additionally, H18Q could also facilitate fusion by aiding in the conformational rearrangements required for exposure of the fusion loop. Combined, the N5R and H18Q mutations might change the binding geometry of suramin, perhaps sidetracking it from the core of the E1/E2 heterodimer/spike and/or facilitating fusion (Figure 4c). 

Our hypothesis that suramin is attracted to the center of domain A is further supported by the observation that the CHIKV variant that is completely dependent on heparan sulfate interactions for infectivity [13,41] has a G82R mutation in E2. In CPE-based assays, the G82R mutant is more sensitive to suramin than wt CHIKV, since 15 µM suramin still gave 85% protection, resulting in an EC_50_ 15 µM for suramin, while the G82R mutation did not influence the EC_50_ for the unrelated compound 6-aza-uridine. Interestingly, residue G82 is located at the center of the spike, where the A domains of the three E2 subunits are found (highlighted in red in Figure 4a), and maps to the area that interacts with Mxra8, a receptor which was recently found to promote CHIKV entry [18]. The introduction of a positive charge at position 82 of E2 could increase the affinity for suramin at the center of the spike and block cell attachment.

These results suggest that suramin could have more impact on the infection when it is attracted to the center of the spike, while more distant positive charges could direct it away from this important region, allowing attachment and interaction with specific receptors, which could explain why the observed the suramin-resistance mutations emerged.

## 4. Conclusions

The aim of our study was to understand how suramin inhibits the early steps of the replicative cycle of CHIKV and other alphaviruses. We have shown that suramin can interact with CHIKV in vitro, and inhibits attachment of the virus to the host cell. Moreover, it could prevent conformational rearrangements in the viral spike glycoproteins that are required for fusion. We were able to select suramin-resistant CHIKV variants and demonstrated that the N5R and H18Q mutations in E2 were responsible for resistance. These mutations did not offer the virus a major advantage during the binding to cells in the presence of suramin. The benefit of these mutations appears to play a role in overcoming suramin’s inhibitory effects during later stages of entry, perhaps allowing the suramin-bound (mutant) spikes to undergo conformational changes required for fusion and progression of the infection. Although CHIKV is able to acquire resistance mutations to the compound, suramin is still an interesting drug candidate as the level of resistance is rather low and required repeated passaging. Additionally, suramin protected human primary dermal fibroblasts from CHIKV-induced CPE with an EC_50_ of approx. 95 µM, proving its efficacy in a more relevant cell model for arbovirus infection.

Regarding its use in humans, suramin could be explored as prophylactic in the context of an outbreak, since it is a compound with one of the longest known half-lives in humans [44]. In previous clinical studies, concerning the treatment of AIDS and certain types of cancer, multiple and serious side effects were attributed to the long-term use of suramin [45]. However, this concerned seriously ill patients and long-term treatment. Such an extended regimen is not required for the treatment of parasitic infections for which suramin has proven to be effective and much better tolerated. Therefore, we believe that also for the short-term treatment or prevention of chikungunya virus infections, suramin would be an interesting drug to evaluate. 

## Figures and Tables

**Table 1:** Mutations in CHIKV resulting from serial passaging in the presence of increasing (suboptimal) concentrations of suramin.

Mutation in	At P5 (150 μM Suramin)		At P7 (300 μM Suramin)	
	nt Substitution	AA Substitution	nt Substitution	AA Substitution
nsP1	G588A	R171Q	G588A A979G G980A	R171Q T301K G302R
nsP3	U5645C	opal524R	ΔU5645-A5650	Δopal524L525
E2	A8554G	N5R	A8554G C8595A	N5R H18Q

**Figure A1:**
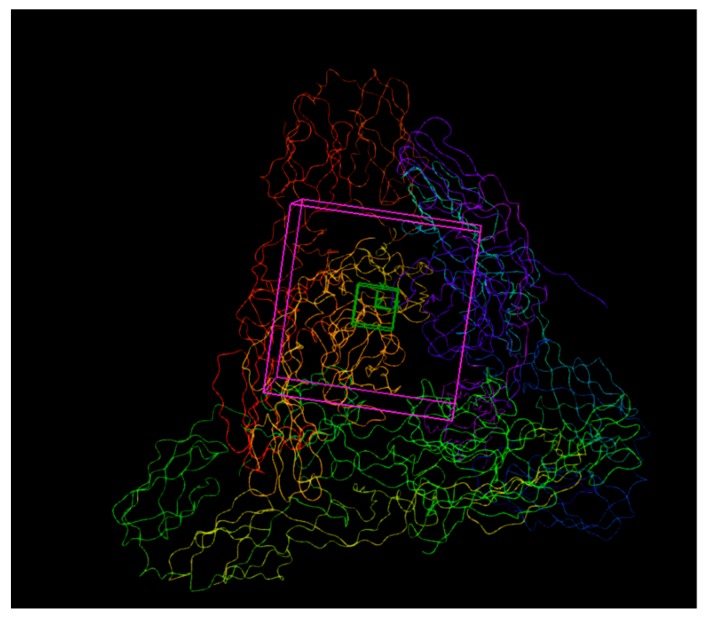
Docking grids used in molecular modeling of the trimeric complex of E1-E2 heterodimers. 36 Å docking grids (inner-box 10 Å and outer-box 46 Å) were prepared as described in the text of Appendix A.

## Supplementary Materials

The following are available online at https://www.mdpi.com/1999-4915/12/3/314/s1, Figure S1: Effect of suramin on the early events of SFV infection, Figure S2: Effect of suramin on CHIKV uptake analyzed by RT-qPCR and Figure S3: The effect of E2 mutations on suramin-resistance determined through a time-of addition assay. Figure S4. Location of F6 and T160 in E2 with respect to a bound molecule of suramin (yellow).

## Appendix A

### Molecular Modeling—In Depth Description of Molecular Modelling

All molecular docking studies were performed on a Viglen Genie Intel^®^CoreTM i7-3770 vPro CPU@ 3.40 GHz × 8 running Ubuntu 14.04. Molecular Operating Environment (MOE) 2018.10 (Chemical Computing Group, 2018) and Maestro (Schrödinger, 2017) were used as molecular modelling software. The CHIKV E2-E1 glycoprotein heterodimer and trimeric complex structures were downloaded from the PDB data bank (http://www.rcsb.org/; PDB code 3N42 and 3J2W). The proteins were preprocessed using the Schrödinger Protein Preparation Wizard by assigning bond orders, adding hydrogens and performing a restrained energy minimization of the added hydrogens using the OPLS_2005 force field. The missing N-terminal residues at the start of the E2 protein (residues 1–6) were manually introduced and energy minimized. Suramin structure was built with MOE and then prepared using the Maestro LigPrep tool by energy minimizing the structures (OPLS_2005 force filed), generating possible ionization states at pH 7 ± 2, generating tautomers and low-energy ring conformers. SiteMap tool in Maestro was used to individuate a potential binding area in proximity of the important mutations, N5R and H18Q. The selected area was used as binding site for the molecular docking studies. For the trimeric complex, three different, one for each heterodimer, 36 Å docking grids (inner-box 10 Å and outer-box 46 Å) were prepared using as centroid a threonine residue localized at the spatial center between the N5 and H18 of two adjacent E2 subunits (Figure A1). In the case of the isolated E2 unit, the same binding area for grid generation was defined selecting as centroid a dummy atom, manually positioned at equal distance from N5 and H18. Molecular docking studies were performed using Glide SP precision keeping the default parameters and setting 5 as number of output poses per input ligand to include in the solution. The output database was saved as mol2 file. The docking results were visually inspected for their ability to bind the active site. The electrostatic potential surface was obtained using the Surfaces and Maps toll in MOE after splitting the molecule in multiple chains. Figures were prepared with MOE.
