# Supplementary material for: Suramin Inhibits Chikungunya Virus Replication by Interacting with Virions and Blocking the Early Steps of Infection
Source: Viruses. 2020 Mar 17;12(3):314. doi: 10.3390/v12030314 (PMC7150963; doi:10.3390/v12030314)
Supplement: Supplementary file 1 [file viruses-12-00314-s001.pdf]

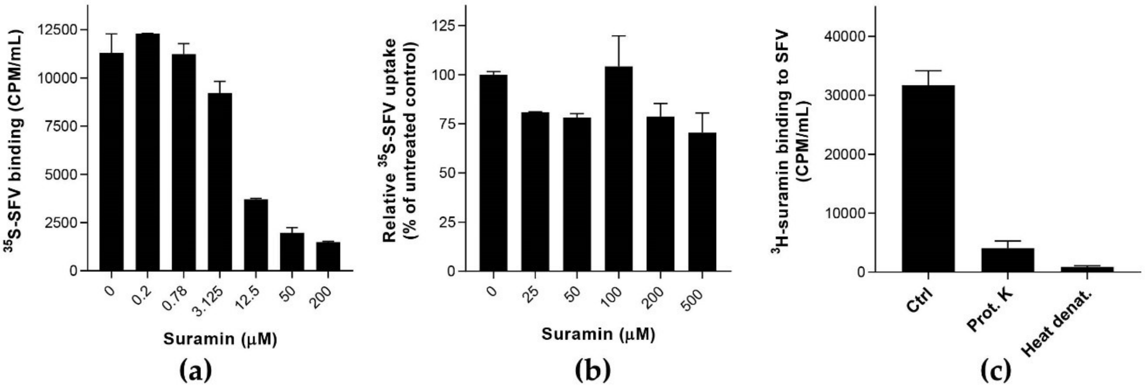

Figure S1. Effect of suramin on the early events of SFV infection. (**a**) Binding of ^{35}S-labelled SFV to Vero E6 cells, at 4°C, in the presence of 4-fold serial dilutions of suramin. (**b**) after a 30-min pre-attachment of virus at 4°C in the absence of the compound, the unbound particles were removed by washing with PBS. Subsequently, medium with suramin was added to the cells followed by incubation at 37°C for 30 min. Cell-associated radiolabeled virus was quantified by cell lysis and liquid scintillation counting. (**c**) ^{3}H-suramin was incubated with purified SFV (in the absence of serum proteins) followed by treatment with proteinase K for 15 min at 37°C or by heat denaturation for 5 min at 95°C, followed by quantification of suramin binding as described for panel B. The average +/- SD is shown; *n* = 2.

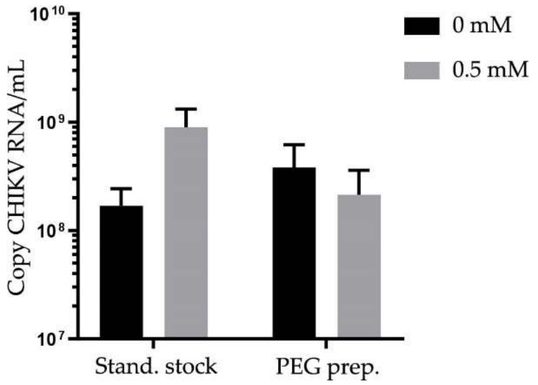

Figure S2. Effect of suramin on CHIKV uptake analyzed by RT-qPCR. The effect of treatment with 0.5 mM suramin during viral uptake (MOI=1) using either a non-purified, standard CHIKV stock (Stand. stock) or a PEG-precipitated stock of the same virus (PEG prec.). After 1h p.i. at 4°C the compound and inoculum (which had been added simultaneously) were washed away with PBS and the CHIKV RNA copy numbers in the cell lysates were analyzed by RT-qPCR (*n* = 2).

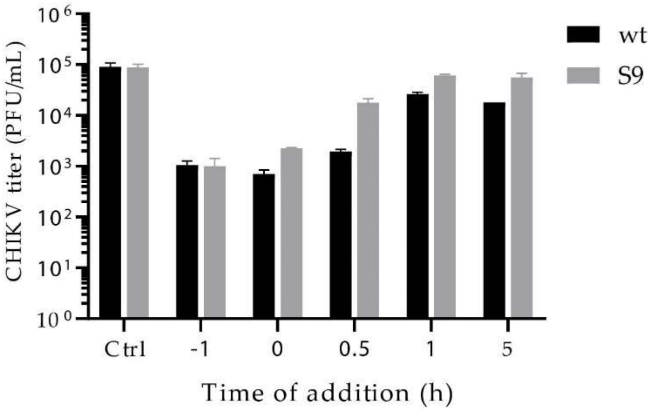

Figure S3. The effect of E2 mutations on suramin-resistance determined through a time-of addition assay. Replication of wt and S9 in the presence of 400 μM suramin, added at various time points prior and after the start of the infection, was determined by measuring the titer of infectious CHIKV at 10 h p.i.

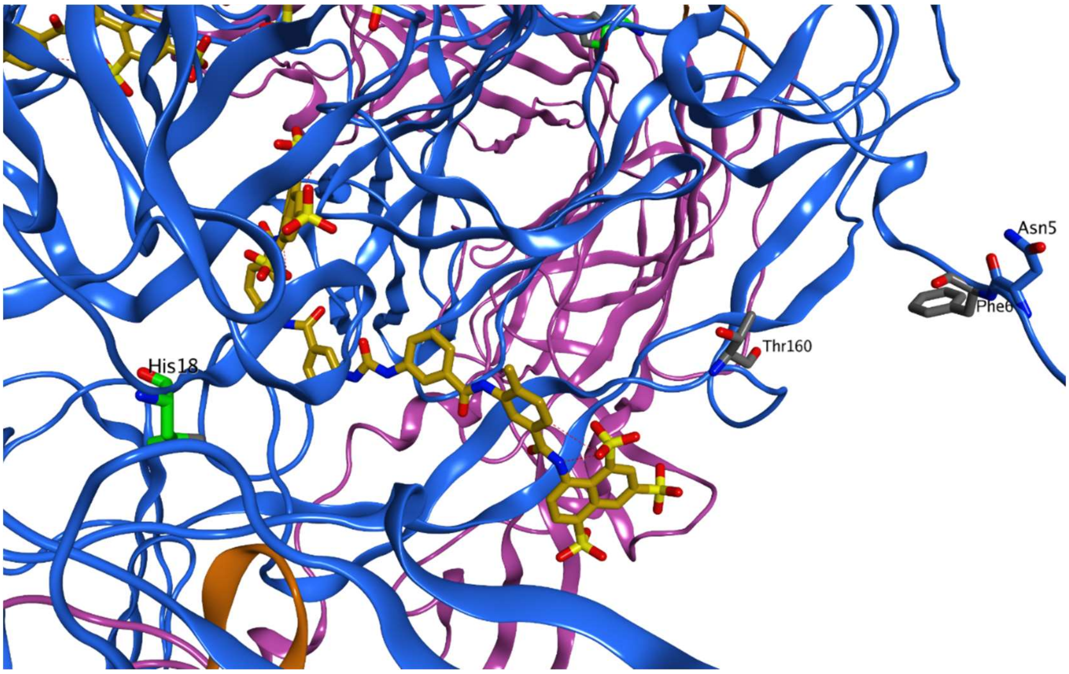

Figure S4. Location of F6 and T160 in E2 with respect to a bound molecule of suramin (yellow). Model based on the structure of a mature CHIKV spike E1-E2 heterotrimer (PDB ID 3J2W). The E2 protein is represented as blue ribbon, E1 as purple ribbon and the fusion loop as orange ribbon. The N5, F6, H18, and T160 residues are indicated.
